# Molecular Basis of Human Sperm Capacitation

**DOI:** 10.3389/fcell.2018.00072

**Published:** 2018-07-27

**Authors:** Lis C. Puga Molina, Guillermina M. Luque, Paula A. Balestrini, Clara I. Marín-Briggiler, Ana Romarowski, Mariano G. Buffone

**Affiliations:** Instituto de Biología y Medicina Experimental, Consejo Nacional de Investigaciones Científicas y Tecnológicas, Buenos Aires, Argentina

**Keywords:** human sperm, capacitation, fertilization, hyperactivation, acrosomal exocytosis

## Abstract

In the early 1950s, Austin and Chang independently described the changes that are required for the sperm to fertilize oocytes *in vivo*. These changes were originally grouped under name of “capacitation” and were the first step in the development of *in vitro* fertilization (IVF) in humans. Following these initial and fundamental findings, a remarkable number of observations led to characterization of the molecular steps behind this process. The discovery of certain sperm-specific molecules and the possibility to record ion currents through patch-clamp approaches helped to integrate the initial biochemical observation with the activity of ion channels. This is of particular importance in the male gamete due to the fact that sperm are transcriptionally inactive. Therefore, sperm must control all these changes that occur during their transit through the male and female reproductive tracts by complex signaling cascades that include post-translational modifications. This review is focused on the principal molecular mechanisms that govern human sperm capacitation with particular emphasis on comparing all the reported pieces of evidence with the mouse model.

## Introduction

In the early 1950s, two researchers, Austin and Chang, using rabbit as a model, independently described the changes that are required for sperm to fertilize oocytes *in vivo* (Austin, 1951; Chang, 1951). These changes were originally grouped under the name of “capacitation” (Austin, 1952) and were later modified to specify that sperm need to reside in the female reproductive tract to acquire this capacity (Austin and Bishop, 1958). These early important observations led to the development of *in vitro* fertilization (IVF). Initially, IVF experiments were performed either with sperm deposited in the oviduct (Austin, 1951; Chang, 1951) or collected from the uterus (Chang, 1959) due to the lack of appropriate conditions to fully support capacitation *in vitro*. A few years later, Yanagimachi and Chang used a medium with a defined chemical composition to capacitate hamster sperm and achieved the first successful IVF (Yanagimachi and Chang, 1963). In 1971, IVF was performed in mice using epididymal sperm and a chemically defined medium (Toyoda et al., 1971).

The remarkable initial discoveries of the fertilization process in mammals were achieved in non-human species such as rabbit, rat, and hamster. The possibility to capacitate mammalian sperm *in vitro* and fertilize the eggs led to the first attempts to capacitate human sperm (Norman et al., 1960; Edwards et al., 1966, 1969). Although little was known about the molecular aspects of human sperm capacitation, these were important steps for achieving the birth of Louise Brown by human IVF (Steptoe and Edwards, 1978).

During capacitation, sperm undergo a change in the motility pattern called hyperactivation (Yanagimachi, 1970) and become competent to undergo a physiological secretory event known as acrosome reaction (aka acrosomal exocytosis; AE). Experiments in mice demonstrated that hyperactivation is critical to fertilization because it facilitates the sperm release from the oviductal reservoir and the penetration through the cumulus oophorus and the extracellular matrix surrounding the egg, i.e., the *zona pellucida* (ZP) (Demott and Suarez, 1992). In addition, mammalian sperm must undergo AE in an orderly manner to penetrate the ZP (Yanagimachi, 1994; Buffone et al., 2009b). It is also proposed that only capacitated human sperm are able to do chemotactic swimming using progesterone gradients in close proximity to the egg (Guidobaldi et al., 2008; Teves et al., 2009; Gatica et al., 2013).

From a molecular point of view, sperm capacitation has been well studied *in vitro* in several species such as bovine, humans, rats, and hamsters, but without any doubt the best characterized model is the mouse. Most of the remarkable discoveries have been generally achieved in mice and later explored in other species. As a scientific tool, mice have helped to speed up the progress of research in all fields, and in sperm physiology, this is true due to several reasons: (i) the possibility to use transgenic tools to create knockout (KO) or transgenic sperm containing fluorescent proteins or molecular sensors; (ii) it is easy to perform assisted reproductive techniques such as intracytoplasmic sperm injection (ICSI), IVF, or embryo transfer; (iii) they are closely related to humans (~99% of mouse genes have an equivalent in humans); (iv) their genome has been fully sequenced (published in 2002); (v) mice are small, have a short generation time, and have an accelerated lifespan; (vi) mice are cost effective because they are inexpensive and easy to look after; (vii) spermatogenesis in mice is comparable with humans (O'Bryan et al., 2006).

Despite the fact that differences might exist between species, mice serve as a *de facto* surrogate model for characterizing the capacitation of human sperm (De Jonge, 2017). However, there are certain aspects that are important to highlight before going deeper into molecular events associated with human sperm capacitation. These considerations not only include differences between both species, i.e., humans and mice, but also important aspects to consider when evaluating *in vitro* experiments. The most significant aspects, according to our opinion, are listed below:

- Human sperm are highly pleomorphic in the sense that a large number of cells in the ejaculate display a great variety of morphological forms. In contrast, the proportion of mouse sperm with morphological variations is rather small.
- Humans deposit the ejaculate in the vagina, in contrast to mice that ejaculate in the uterus (Kawano et al., 2014).
- Human sperm are selected in the cervix, where only morphologically normal or slightly abnormal sperm can migrate through this channel. A cohort of sperm immediately pass into the cervical mucus, whereas the remaining sperm population becomes a part of the coagulum. Then, a second round of selection occurs in the uterotubal junction (UTJ). In contrast, mouse sperm are only selected in the UTJ by mechanisms that are not fully clarified but include ADAM3 and other proteins (Yamaguchi et al., 2009; Holtzmann et al., 2011; Okabe, 2013).
- In general, the study of human sperm starts from a semen sample, whereas in mice, it starts from sperm recovered from the epididymis. In this condition, mouse sperm has not yet been exposed to high concentrations of ${\text{HCO}}_{3}^{-}$, cholesterol, Zn^2+^, and seminal plasma proteins, among other components.
- In humans, the semen is frequently manipulated to isolate the highly motile population of sperm. In contrast, virtually all studies use mouse sperm obtained from the cauda epididymis that have not been exposed to any selection procedure.
- *In vitro* incubation under capacitating conditions for human sperm ranges from 3 to 24 h. As a result, a great variability of results is reported in the literature. In contrast, most studies in mouse sperm are performed using 1–1.5 h of incubation under capacitating conditions.
- Based on non-human data, the oviductal epithelium is considered a sperm reservoir that regulates binding and release of sperm toward the site of fertilization. The role of oviductal epithelium and fluids on human sperm was generated *in vitro* by cell culture experiments. Hence, our knowledge about human sperm interaction with the oviduct is scarce in comparison with rodents.
- The role of the uterus, the oviduct, and their secretions on human sperm capacitation is largely unknown due to practical and ethical limitations (De Jonge, 2017). A great number of molecules that are present in the female tract that have also been shown to modify sperm function are usually not included in the *in vitro* capacitation experiments (Luconi et al., 1995; Meizel et al., 1997; Edwards et al., 2007; Garbarino Azúa et al., 2017). In addition, uterine contractions facilitate the sperm transport mechanism that is essential for migration within the female reproductive tract.

For all these reasons (and many others that will be explained in the following sections), caution while transferring molecular and cellular concepts between species was proposed recently (Kaupp and Strünker, 2016). Alternatively, sperm from a given species should be studied using a vertical research strategy (Kaupp and Strünker, 2016).

We would also like to stress that, unless otherwise indicated, all data regarding human sperm function and regulation by electrophysiological processes are derived from *in vitro* experimentation and may not be reflective of what occurs during transit through the male and female reproductive tracts. The aim of this paper is to revisit the most important molecular events of human sperm capacitation.

## Sperm plasma membrane and seminal plasma cholesterol

The sperm plasma membrane not only serves as the cell boundary but also presents a dynamic structure that has an impact on sperm capacitation and AE (Flesch and Gadella, 2000). During capacitation, several changes in the sperm membrane have been described: increase in membrane fluidity, lateral movement of cholesterol to the apical region of the sperm head, and cholesterol efflux from the sperm plasma membrane to the extracellular environment (Martínez and Morros, 1996; Gadella, 2008). The approximate lipid content of mammalian sperm is composed of 70% phospholipids, 25% neutral lipids (cholesterol), and 5% glycoproteins (Mann and Lutwak-Mann, 1981), cholesterol being the main sterol in the cellular plasma membrane (~90%) (Lalumière et al., 1976; Langlais et al., 1981; Zalata et al., 2010; Boerke et al., 2013). In addition, desmosterol, a cholesterol precursor, and sulfate derivatives were reported (~10%) (Nimmo and Cross, 2003).

The cholesterol/phospholipid (C/PL) ratio in sperm varies between species (Davis, 1981), i.e., 0.20 in boar sperm, 0.36 in stallion sperm, about 0.40 in bovine sperm, 0.43 in ram sperm, and 0.83 in human sperm (Parks and Hammerstedt, 1985; Parks et al., 1987, 1992). Davis reported a correlation between the C/PL ratio in sperm and the time required to complete capacitation when comparing different mammalian species: the higher the C/PL ratio, the longer the incubation period for capacitation to be achieved (Davis, 1981; Ostermeier et al., 2018). Sperm of patients with unexplained infertility showed a higher C/PL ratio due to lower phospholipid content (Sugkraroek et al., 1991), and normospermic patients who failed in IVF had either an atypical high content of cholesterol or a slow efflux of cholesterol during *in vitro* incubation (Benoff et al., 1993).

Sperm cholesterol content is finely regulated within the male reproductive tract as the concentration of lipids in blood serum does not correlate with the seminal plasma levels (Grizard et al., 1995). Cholesterol is found in high abundance in seminal plasma (Grizard et al., 1995; Cross, 1996). Experiments in rabbit and bull sperm showed an inhibitory effect of seminal plasma on capacitation that could be reversed after re-incubation of the sperm in the oviduct (Chang, 1957). Incubation of human sperm in seminal plasma inhibited progesterone-induced AE, being the main inhibitor free cholesterol (Cross, 1996). Altogether, these early observations demonstrate the important regulatory role of seminal plasma sterols on the initiation and promotion of capacitation.

### Cholesterol efflux during capacitation

It has been well demonstrated *in vitro* that capacitation is associated with removal of cholesterol from the plasma membrane (Visconti et al., 1999). Albumin is the most used cholesterol acceptor in *in vitro* experiments (Langlais et al., 1988; Suzuki and Yanagimachi, 1989; Leahy and Gadella, 2015), and it has been described to be in high abundance in the oviduct (Ehrenwald et al., 1990). The lipid transfer protein-I (LTP-I), a key protein in the human plasma metabolism of the high-density lipoprotein (HDL) (Albers et al., 1984; Tall, 1993), is present in the reproductive fluids and it also serves as a cholesterol acceptor (Ravnik et al., 1992).

Sterol-rich microdomains, known as lipid rafts, are organization centers involved in membrane protein distribution, activating receptors and signaling cascades. Markers for these rafts, such as the proteins caveolin-1, caveolin-2, flotilin-1 and flotilin-2, and the sphingolipids GM1 and GM3, have been described (Travis et al., 2001; Suzuki et al., 2017). A capacitation-associated movement, due to cholesterol efflux, of GM1 has been observed during capacitation (Selvaraj et al., 2007; Bruckbauer et al., 2010). GM1 binds decapacitating factors released during capacitation (Kawano et al., 2008) and can be used as a biomarker for lipid rafts, as it can be easily traced using cholera toxin (Selvaraj et al., 2006).

Lipocalin 2 is present in mouse oviduct and uterus and induces capacitation via raft aggregation in a PKA-dependent manner (Watanabe et al., 2014). Glycosylphosphatidylinositol-anchor proteins (GPI-APs) are also components of lipid rafts (Varma and Mayor, 1998), and their release is very important for male fertility (Kondoh et al., 2005; Ueda et al., 2007; Fujihara et al., 2013). Recent studies in mouse sperm described the importance of lipid raft movement in order for sperm to gain fertilization ability, using cholera toxin to track GM1 and (GPI)-anchored enhanced green fluorescent protein (EGFP-GPI) (Kondoh et al., 1999; Watanabe et al., 2017). Cholesterol efflux using methyl-β-cyclodextrin (M-β-CD) showed not only GM1 movement but also release of GPI-APs (Watanabe et al., 2017).

Phospholipid scrambling, one of the earliest capacitation events, is initiated by an increase in intracellular ${\text{HCO}}_{3}^{-}$ followed by the activation of the cAMP/PKA pathway and may be essential to facilitate albumin-mediated cholesterol efflux (Gadella and Harrison, 2000; Harrison and Miller, 2000; Flesch et al., 2001).

Ravnik and coworkers proposed LTP-I as a capacitation inducer in human sperm, as it stimulates acrosomal loss and increases the penetration of hamster eggs by human sperm (Ravnik et al., 1995).

## Activation of cAMP-PKA pathway

Human sperm capacitation can be mimicked *in vitro* in a chemically defined medium containing electrolytes (Na^+^, K^+^, Cl^−^, ${\text{HCO}}_{3}^{-}$, Mg^2+^, Ca^2+^, and ${\text{PO}}_{4}^{3-}$), energy substrates (glucose, pyruvate, and lactate), and a cholesterol acceptor (usually serum albumin as previously described).

The activation of intracellular signaling pathways is dependent on the presence of the chemicals present in the capacitation medium. For instance, once human sperm are exposed to seminal plasma or the female reproductive tract, they encounter higher concentration of ${\text{HCO}}_{3}^{-}$ (Okamura and Sugita, 1983; Okamura et al., 1985), which in turn stimulates the soluble adenylyl cyclase ADCY10 (Buck et al., 1999; Jaiswal and Conti, 2003). Activation of ADCY10 primarily by ${\text{HCO}}_{3}^{-}$, but also by Ca^2+^ leads to an increase in cyclic adenosine monophosphate (cAMP) synthesis in several mammalian species (Chen et al., 2000). The initial ${\text{HCO}}_{3}^{-}$ entrance in mouse and human sperm occurs through NBC cotransporters (Demarco et al., 2003; Puga Molina et al., 2018). In addition, it was reported that inhibition of the cystic fibrosis transmembrane conductance regulator channel (CFTR) affects ${\text{HCO}}_{3}^{-}$-entrance-dependent events (Puga Molina et al., 2017), such as phosphorylation in substrates of protein kinase A (PKA) and tyrosine phosphorylation (pY). In contrast, CFTR inhibition does not affect this pathway in mouse sperm (Wertheimer et al., 2008; Puga Molina et al., 2017).

In sperm as well as in other cells, intracellular cAMP levels are highly dynamic. Its concentration relies on the simultaneous action of both synthesis by ADCY10 and degradation by phosphodiesterases (PDE). In mammals, 11 PDE families have been described, with different substrate specificities and pharmacological sensitivities. In human sperm, inhibition of PDE4 enhanced sperm motility, whereas PDE1 inhibitors selectively stimulated the AE (Fisch et al., 1998). These observations suggest that molecules related to cAMP signaling such as cAMP targets, adenylyl cyclases, and PDE are compartmentalized and, as a consequence, participate in different sperm functions, some in the flagellum and others in the head (Buffone et al., 2014b).

The role of cAMP in sperm function is well described elsewhere (Buffone et al., 2014b), and some of its targets will be discussed in the following sections. One of the main targets of cAMP is PKA, which is essential in sperm biology (Burton and McKnight, 2007). PKA is an heterotetramer composed of two catalytic subunits (C) and two regulatory subunits (R). The active C subunit is dissociated as an active kinase when cAMP binds to R subunits. Using antibodies against PKA substrate consensus phosphorylation sites, it was shown in human sperm as well as in other species that PKA activity reaches maximum activity within 1 min of exposure to ${\text{HCO}}_{3}^{-}$ (Battistone et al., 2013). Because PKA has multiple targets, phosphorylation of a given substrate may occur without affecting others by the action of A-kinase-anchoring proteins (AKAPs) (Carnegie et al., 2009). AKAPs anchor the R subunit of PKA, restricting its activity to discrete locations within the sperm (Carnegie et al., 2009; Scott and Pawson, 2009). Several reports have shown the presence and possible function of AKAPs such as AKAP3 and AKAP4 in human sperm (Carrera et al., 1996; Mandal et al., 1999; Harrison et al., 2000; Ficarro et al., 2003). In addition to PKA, cAMP can bind and regulate other targets such as the exchange protein directly activated by cAMP (EPAC). EPAC1 and EPAC2 are expressed in sperm from different species including human (Branham et al., 2006) and are localized to the sperm head. These enzymes play a major role in human sperm AE (Branham et al., 2006, 2009; Buffone et al., 2014a).

One of the best characterized events in sperm capacitation is the time-dependent increase in pY. The increase in sperm pY is downstream of a cAMP/PKA-dependent pathway in many species including humans (Visconti et al., 1995b; Osheroff et al., 1999; Battistone et al., 2013, 2014). Several reports have shown clear deficiencies in this process in infertile patients (Buffone et al., 2004, 2005, 2006, 2009a,c). Because PKA is a serine/threonine (Ser/Thr) protein kinase, a tyrosine kinase mediates the role of PKA in pY. The mechanism by which PKA activates pY in humans was reported to be mediated by proline-rich tyrosine kinase 2 (PYK2) (Battistone et al., 2014). On the contrary, sperm from *Pyk2*^−/−^ mice have normal pY during capacitation, but in sperm from mice in which the tyrosine kinase FER was disrupted, pY was not increased (Alvau et al., 2016). FER has also been detected in human sperm (Matamoros-Volante et al., 2017), although its role during capacitation has not yet been established.

In summary, the cAMP/PKA signaling pathway is essential for human sperm capacitation and is activated by ${\text{HCO}}_{3}^{-}$ and Ca^2+^ influx during the sperm transit from the epididymis to the oviduct. During this journey, sperm are exposed to large changes in ${\text{HCO}}_{3}^{-}$, Ca^2+^, as well as H^+^, Na^+^, K^+^ that ultimately impact on the membrane potential (Em) and the intracellular pH. These changes are regulated by the activation of the cAMP-PKA pathway and they will be explained in detail in the following sections.

## Extracellular and intracellular pH in human sperm

Regulation of intracellular pH (pHi) is fundamental for every cellular process. It is suggested that homeostasis of the pHi in mammals is mainly controlled by: (1) H^+^ and (2) ${\text{HCO}}_{3}^{-}$ transport. Particularly, sperm encounter a variety of dramatic changes in H^+^ extracellular concentration during their transit from the epididymis to the site of fertilization in the female tract. Although extracellular pH (pHe) from epididymis is acidic (approx. 6.8) (Carr and Acott, 1989; Caflisch and DuBose, 1990; Rodriguez-Martinez et al., 1990), in humans, the pH of semen is approximately 7.2–8.4 (Owen and Katz, 2005), and in the human female, the reproductive tract is graduated, with lowest pH in the vagina (approx. pH 4.4), increasing toward the endocervix and uterus (approx. pH 7) (Macdonald and Lumley, 1970; Eggert-Kruse et al., 1993; Ng et al., 2017).

In addition to different H^+^ concentrations, sperm encounter a variety of different ionic compositions such as ${\text{HCO}}_{3}^{-}$. In the porcine epididymis, the [${\text{HCO}}_{3}^{-}$]_e_ is approximately 2–4 mM (Okamura et al., 1985), whereas in rabbit, human, and porcine, seminal plasma is approximately 25 mM (Vishwakarma, 1962), and in the human and rabbit female tract, it is reported in the range of approximately 20–60 mM (Vishwakarma, 1962; Hamner et al., 1964; David et al., 1973).

In addition, it is postulated that pHe varies in the female tract according to the moment of ovulation. In the lumen of the *Macaca mulatta* (rhesus monkey) oviduct, pHe increases from approximately 7.2 to 7.6, whereas the [${\text{HCO}}_{3}^{-}$]_e_ increases from approximately 35 to 90 mM from the follicular phase to ovulation (Maas et al., 1977). These variations in [H^+^]_e_ and [${\text{HCO}}_{3}^{-}$]_e_ during the journey of the sperm and during ovulation in the female tract might be of great importance for the pHi regulation in sperm.

### Alkalinization during capacitation

During their transit through the female reproductive tract, sperm encounter an alkaline pH, higher ${\text{HCO}}_{3}^{-}$ concentration, and albumin. All these factors contribute to the cytoplasmic alkalinization that occurs during mouse sperm capacitation (Zeng et al., 1996; Nishigaki et al., 2014). This event is widely associated with hyperactivated motility because the alkalinization of the cytoplasm is necessary for the activation of CatSper, and the activity of this channel is fundamental for the hyperactivation of the human sperm (see below).

In mouse sperm, it was shown that sperm alkalinization depends mainly on the Na^+^/H^+^ exchanger (NHE) activity (Wang et al., 2003b; Chávez et al., 2014) and also on the CFTR activity (Xu et al., 2007; Chávez et al., 2012). However, in humans, the mechanism of the pH increase is thought to be different (Miller et al., 2016) as it is postulated that the main proton efflux depends mostly on Hv1 (Lishko et al., 2010).

The pHi of mammalian sperm, including humans, has been evaluated using different fluorescent indicators (Florman et al., 1989; Vredenburgh-Wilberg and Parrish, 1995; Brook et al., 1996; Hamamah et al., 1996; Cross and Razy-Faulkner, 1997), [^31^P]-NMR (Smith et al., 1985; Robitaille et al., 1987), and also by the distribution of a radioactive amine (Gatti et al., 1993; Hamamah et al., 1996), resulting in pHi approximately 6.7–7.2. However, there are few reports showing an increase in pHi during capacitation in human sperm. By using the BCECF pH-sensitive fluorescent probe, Cross and Razy-Faulkner showed that 24-h capacitated sperm have higher pHi (7.08) compared with freshly ejaculated sperm (6.94). They also showed that when cholesterol loss is prevented, pHi is similar to that observed in ejaculated sperm (pHi approx. 6.7) (Cross and Razy-Faulkner, 1997). López-González et al. demonstrated by flow cytometry the existence of a subpopulation of capacitated sperm with more alkaline pH than those incubated in a noncapacitating medium (López-González et al., 2014). Because of the lack of *in vivo* experimentation, overall, the alkalization as a regulatory process during human sperm capacitation is still highly speculative.

### Regulation of pHi

Although alkalinization has been demonstrated in human sperm *in vitro*, the molecular mechanisms related to this process have not yet been fully understood, and still remains much to be explored regarding the participation of different channels and transporters during capacitation. As mentioned before, ion transporters that regulate pHi can be divided into two groups: (1) H^+^ transporters and (2) ${\text{HCO}}_{3}^{-}$ transporters.

#### Voltage-gated H^+^ channels (Hv1)

Hv1 is encoded by the *HVCN1* gene and mediates highly selective H^+^ outward currents (Musset and Decoursey, 2012). Hv1 is the dominant proton conductance in human sperm; however, until now, the effect on Hv1 mutations in human fertility has not been reported. In contrast, mouse sperm do not have functional Hv1 (Lishko and Kirichok, 2010), and for this reason, Hv1^−/−^ mice are fertile (Ramsey et al., 2009).

The Hv1 channel is present in the principal piece of the flagellum of human sperm as confirmed by immunoblotting and immunostaining (Lishko et al., 2010), and recently, a shorter variant (Hv1Sper) generated by proteolytic cleavage during spermatogenesis was reported (Berger et al., 2017).

Electrophysiological data have shown that Hv1 is an H^+^-selective channel whose activity is potentiated by capacitation, anandamide, membrane depolarization, and alkaline extracellular pH (Lishko et al., 2010). Interestingly, this channel is inhibited by Zn^2+^ (IC50 = 222 ± 36 nM) (Lishko et al., 2010; Qiu et al., 2016), which is present in high concentration in seminal plasma [in humans approx. 1.2–10.6 mM in seminal fluid vs. approx. 15.3 μM in serum (Owen and Katz, 2005)]. Hv1Sper is also inhibited by Zn^2+^, but the loss of a fragment in Hv1 N-terminus tunes its sensitivity to pH. Hv1Sper variant can form heterodimers with Hv1. Hv1Sper-Hv1 tandem dimers display distinct pH and voltage dependence; however, the Hv1Sper/Hv1 ratio is independent of capacitation (Berger et al., 2017).

Although it has been proposed that Hv1 would be mainly responsible for pH control in human sperm, the participation of this channel on the rise of pHi during capacitation has not been reported yet.

#### Na^+^/H^+^ Exchangers (NHE)

The SLC9 gene family encodes 13 evolutionarily conserved NHE. The expression of three NHEs has been identified in rat, mouse and human sperm, such as NHE1, NHE5, and NHE10 (Woo et al., 2002; Wang et al., 2003a; Zhang et al., 2017). Furthermore, in mouse sperm, a new member of the NHE family (sperm-specific NHE; sNHE, *Slc9c1* gene) is expressed, whose localization is restricted to the principal piece (Wang et al., 2003a). sNHE-null males are infertile and have impaired sperm motility. As sNHE not only interacts but is also required for the sAC expression, it is postulated that this complex modulates pHi and ${\text{HCO}}_{3}^{-}$ (Wang et al., 2007). Regarding the participation of pHi and ${\text{HCO}}_{3}^{-}$ in sperm motility, it is worth knowing that the addition of ${\text{NH}}_{4}^{+}$ and cAMP analogs partially rescues the motility and fertility defects, suggesting that other important players may also be affected in this transgenic model (Wang et al., 2003a).

In human sperm, sNHE is mainly localized in the principal piece and its expression is downregulated in sperm from asthenozoospermic patients (Zhang et al., 2017). In addition, it has been reported that regulation of pHi in human sperm depends on the [Na^+^]_e_, and that ethyl-isopropyl amiloride (EIPA) affects this regulation within concentrations that inhibit NHE activity (Garcia and Meizel, 1999). Amiloride, another inhibitor of NHE, at 0.5 mM affects motility in human sperm, and the addition of nigericin, an ionophore that restores intracellular pH, partially rescues sperm motility (Peralta-Arias et al., 2015). As in mouse sperm, the ion transport-like region of the putative human sNHE is related to the membrane segments of voltage-gated ion channels (Wang et al., 2003a). For this reason, it is suggested that sNHE should play a central role in signaling (Kaupp and Strünker, 2016). Unfortunately, because sNHE is electroneutral, it is difficult to use traditional electrophysiological techniques to study its role in human sperm (Miller et al., 2015), and its role during capacitation still remains to be elucidated.

#### ${\text{HCO}}_{3}^{-}$ transporters

As ${\text{HCO}}_{3}^{-}$ is a weak base, changes in [${\text{HCO}}_{3}^{-}$]_i_ can cause intracellular alkalinization. ${\text{HCO}}_{3}^{-}$ transporters include the SLC26 and SLC4 families (Liu et al., 2012) and the CFTR (Anderson et al., 1991).

- **SLC4:** SLC4A1–5 and SLC4A7–11 family members include two groups: an Na^+^-independent group and an Na^+^-dependent group (Liu et al., 2012; Bernardino et al., 2013). Na^+^-independent members include three anion exchangers, namely SLC4A1 (AE1), SLC4A2 (AE2), and SLC4A3 (AE3), which mediate electroneutral Cl^−^/${\text{HCO}}_{3}^{-}$ exchange (Holappa et al., 1999; Medina et al., 2003). Although in human sperm, the presence of SLC4A1 and SLC4A2 in the equatorial segment has been demonstrated (Parkkila et al., 1993), the participation of this family in the regulation of pHi is unknown. The Na^+^-dependent members of the SLC4 family include five Na^+^-coupled ${\text{HCO}}_{3}^{-}$ transporters, also termed NBC. The NBC transporters are composed of two electrogenic Na^+^/${\text{HCO}}_{3}^{-}$ cotransporters, NBC1 (SLC4A4, 2 ${\text{HCO}}_{3}^{-}$:1 Na^+^) and NBC2 (SLC4A5, 2 ${\text{HCO}}_{3}^{-}$:1 Na^+^), two electroneutral Na^+^/${\text{HCO}}_{3}^{-}$ cotransporters, NBCn1 (SLC4A7; 2 ${\text{HCO}}_{3}^{-}$:1 Na^+^) and NBCn2 (SLC4A10), and an electroneutral Na^+^-driven Cl^−^/${\text{HCO}}_{3}^{-}$ exchanger, NDCBE (SLC4A8 2 ${\text{HCO}}_{3}^{-}$:1 Na^+^). The Na^+^ dependence of “AE4” (SLC4A9) remains controversial (Liu et al., 2015); however, the latest evidence suggests that it is an electroneutral Cl^−^/nonselective cation–${\text{HCO}}_{3}^{-}$ exchanger (Peña-Münzenmayer et al., 2016).
  Na^+^-coupled ${\text{HCO}}_{3}^{-}$ transporters have been shown to play a role in the regulation of pHi during capacitation. Demarco et al. (2003) suggested that an electrogenic NBC is active in mouse sperm and is responsible for the initial ${\text{HCO}}_{3}^{-}$ entrance during capacitation. In addition, in mouse sperm, Zeng et al. (1996) demonstrated that pHi is dependent on extracellular Na^+^, ${\text{HCO}}_{3}^{-}$, and Cl^−^. Jensen et al. (1999) showed the expression of the NBC1 in rat sperm. In humans, NBC2, NDCBE, and NBCn2 were detected in testis (Ishibashi et al., 1998; Damkier et al., 2007). It has recently been shown that NBC is involved in the initial ${\text{HCO}}_{3}^{-}$ uptake in humans (Puga Molina et al., 2018).

- **SLC26:** In mouse and human sperm, elevation of pHi was shown to depend on CFTR activity (Xu et al., 2007; Puga Molina et al., 2017). It has also been shown that there is a physical interaction between CFTR and the SLC26A3, SLC26A6, and SLC26A8 exchangers in mouse, human, and guinea pig sperm (Chen et al., 2009; Chávez et al., 2012; Rode et al., 2012). This functional association between CFTR and the SLC26A3 and SLC26A6 modulates pHi in mouse sperm (Chávez et al., 2012).
  CFTR is a selective ion channel to Cl^−^ (Anderson et al., 1991; Bear et al., 1992; Tabcharani et al., 1993) and also transports other anions with different permeabilities (pBr^−^ ≥ pCl^−^ > pI^−^ > p${\text{HCO}}_{3}^{-}$) (Anderson et al., 1991). This ATP-gated channel is regulated by PKA, because its phosphorylation is mandatory for both the channel opening mechanism and the ATP association (Anderson et al., 1991; Tabcharani et al., 1991; Bergerz et al., 1993). The multiple potential sites of phosphorylation by PKA in the regulatory domain of CFTR (R) make the channel dependent on the cAMP concentration (Tabcharani et al., 1991; Bergerz et al., 1993; Sheppard and Welsh, 1999; Gadsby et al., 2006; Sorum et al., 2015). In addition, the interaction between CFTR and SLC26 is mediated by the R domain of the channel and the Sulfate Transporter and Anti-Sigma (STAS) domain of SLC26, which must be phosphorylated by PKA to favor interaction (Gray, 2004; Ko et al., 2004). In accordance to these results, it was reported by our group that PKA activity is essential for pHi regulation in human sperm (Puga Molina et al., 2017).
  CFTR protein is present in mature human and mouse sperm and is restricted to the mid-piece (Hernández-González et al., 2007; Xu et al., 2007) and the equatorial segment of the head (Xu et al., 2007).
  In humans, mutations in the CFTR gene that impair CFTR activity cause a severe disease called cystic fibrosis. It has been reported that patients with cystic fibrosis have deterioration in fertility in both women and men. The higher incidence of CFTR mutations in a male infertile subpopulation may indicate its participation in other fertilization-related events, such as sperm capacitation (Jakubiczka et al., 1999; Schulz et al., 2006). Supporting this hypothesis, human sperm treated with a specific inhibitor of CFTR decreases the percentage of sperm undergoing AE, hyperactivation, and penetration of ZP-free hamster eggs (Li et al., 2010). Regarding the SLC26 transporters that can interact in human sperm with CFTR, SLC26A6 is expressed in human efferent and epididymal ducts and colocalizes with CFTR (Kujala et al., 2007). SLC26A8 (TAT1) is expressed specifically in the male germ line, and it was shown to physically interact with CFTR *in vitro* and *in vivo* in mature sperm, activates CFTR, and, interestingly, is essential for the activation of the cAMP-PKA pathway in mouse sperm (Rode et al., 2012). Its role in human sperm has not been demonstrated but nonsense mutations in *SLC26A8* have been associated with asthenozoospermia (Dirami et al., 2013). In addition, in humans, mutations that impair SLC26A3 activity also cause subfertility and oligoasthenozoospermia (Hihnala et al., 2006; Höglund et al., 2006).

#### Carbonic anhydrases (CAs)

Carbonic anhydrases (CAs) are metalloenzymes that catalyze the reversible hydration of carbon dioxide to ${\text{HCO}}_{3}^{-}$ (OH^−^ + CO_2_ ↔ ${\text{HCO}}_{3}^{-}$ + H^+^). CAs are encoded by five gene families: α, β, ɤ, δ, and ζ, but only 15 isoforms of the α family are found in primates (i.e., CAI-CAXIV, except CAXV) (Truppo et al., 2012). CAs are important in the regulation of pHi in bacteria, archaea, and eukarya. However, the role of these enzymes in sperm physiology is still not clear (Nishigaki et al., 2014). The expression of some CAs has been reported in human sperm, including CAI (Ali Akbar et al., 1998) and CAII (Ali Akbar et al., 1998), that were reported in the post-acrosomal region (Parkkila et al., 1991) and in the flagellum (José et al., 2015), and CAXIII (Lehtonen et al., 2004) localized in the flagellum of human sperm (José et al., 2015). The function of CAs is not yet fully understood, but the use of general blockers against these enzymes affects motility and increases the AE in capacitated human sperm (Wandernoth et al., 2010; José et al., 2015).

#### Albumin

Cross (1998) demonstrated that the cytoplasmic alkalinization in human sperm depends on the cholesterol removal during capacitation. Cross and Razy-Faulkner (1997) showed that sperm cells incubated with albumin saturated with cholesterol sulfate have a more acidic pHi than the capacitated control condition. Although the effects of cholesterol in pHi have been observed in platelets and fibroblasts (Poli de Figueiredo et al., 1991) and that cholesterol alters the activity of NHE and Cl^−^/${\text{HCO}}_{3}^{-}$ exchangers in erythrocytes (Grunze et al., 1980), how this regulation occurs in human sperm remains largely unknown.

The proton-selective voltage-gated channel Hv1 can be activated by removing extracellular Zn^2+^ (Lishko and Kirichok, 2010; Lishko et al., 2010). It has been proposed that high concentrations of Zn^2+^ in the seminal plasma inhibit Hv1, and that in the female tract a decrease in Zn^2+^ due to dilution, absorption by the uterine epithelium, and chelation may render sperm free from Zn^2+^ in the fallopian tube (Gunn and Gould, 1958; Ehrenwald et al., 1990; Lu et al., 2008). Albumin is not only a cholesterol acceptor but it also chelates Zn^2+^ (Lu et al., 2008). Therefore, it is unclear whether the effect on pHi is due to the cholesterol efflux and/or the chelation of Zn^2+^.

## Membrane potential in human sperm

In any given cell, the metabolic state and specific ion channels and transporters determine the internal and the external ion concentration and the plasma membrane permeability that defines the Em. Sperm encounter different concentrations of extracellular K^+^, Na^+^, Cl^−^, and ${\text{HCO}}_{3}^{-}$ throughout their journey from the testis to the site of fertilization in the female tract. In the *ductus deferens*, the levels of K^+^ (approx. 110 mM), Na^+^ (approx. 30 mM), Cl^−^ (approx. 100 mM) (Hinton et al., 1981) (for humans), and ${\text{HCO}}_{3}^{-}$ (approx. 2–4 mM) (Okamura et al., 1985) (for porcine) are different than in seminal plasma [K^+^ (approx. 12–63 mM), Na^+^ (approx. 102–143 mM), Cl^−^ (approx. 37–45 mM), and ${\text{HCO}}_{3}^{-}$ (approx. 25 mM)] (Okamura et al., 1986; Owen and Katz, 2005), and than in human uterine tubal fluid [K^+^ (approx. 4.5–21 mM), Na^+^ (approx. 130–149 mM), Cl^−^ (approx. 118–132 mM), and ${\text{HCO}}_{3}^{-}$ (approx. 20 a 60 mM)] (Lippes et al., 1972; David et al., 1973; Lopata et al., 1976; Borland et al., 1977; Aguilar and Reyley, 2005). Although Na^+^ and ${\text{HCO}}_{3}^{-}$ are higher in seminal plasma and in the female tract than in the *ductus deferens*, K^+^ is lower and Cl^−^ varies reaching maximal concentration in the uterine tubal fluid. These ionic changes transduce variations not only in the Em but also in pH, as mentioned earlier.

In human sperm, it was demonstrated that the regulation of Em is related to male fertility due to the modulation of ion channels and transporters such as CatSper (sperm-specific Ca^2+^ channel) and Hv1 (Darszon et al., 1999; Lishko et al., 2012). It was reported that idiopathic and asthenozoospermic infertile men have more depolarized Em than fertile men (Calzada and Tellez, 1997), and that depolarization of Em is associated with low IVF success rate in subfertile men (Brown et al., 2016).

### Hyperpolarization during capacitation

Hyperpolarization of the Em occurs when there is an increase in the concentration of net negative charges in the intracellular compartment. Membrane hyperpolarization during capacitation has been demonstrated in murine, bovine, equine, and human sperm (Zeng et al., 1995; Hernández-González et al., 2007; Escoffier et al., 2012; López-González et al., 2014). Experiments in mouse sperm demonstrate that hyperpolarization is necessary and sufficient to prepare them for AE (De La Vega-Beltran et al., 2012).

Compared with mouse sperm, it was reported that changes in Em in human sperm are not as evident, probably due to the variability between donors and the small difference in Em values between capacitated and noncapacitated sperm. This could be because changes in the Em occur in a small fraction of human sperm population (López-González et al., 2014). Therefore, methods such as flow cytometry to distinguish membrane hyperpolarization are useful for studying this event. The reported values of resting Em in noncapacitated human sperm are approximately −40 mV (Linares-Hernández et al., 1998) and approximately −17.7 mV (Brown et al., 2016). In capacitated human sperm, these values shift to approximately −58 mV (Patrat et al., 2002) and approximately −22.7 mV (Brown et al., 2016). The differences between these values may be methodological: although Brown et al. inferred resting Em from reversal potential obtained by whole cell patch clamping, Linares-Hernández and Patrat used fluorimetry. In mouse sperm, the resting Em of noncapacitated sperm is approximately −35 to −45 mV, and after capacitation, this value changes to approximately −65 mV (Espinosa and Darszon, 1995; Zeng et al., 1995; Muñoz-Garay et al., 2001; Demarco et al., 2003; Hernández-González et al., 2006; Santi et al., 2010; De La Vega-Beltran et al., 2012).

### Regulation of Em

It was reported that hyperpolarization of the plasma membrane occurs downstream of cAMP elevation in mouse and human sperm (Martínez-López et al., 2009; Escoffier et al., 2015; Puga Molina et al., 2017). In mouse sperm, it was suggested that cSrc is activated downstream of PKA and modulates the sperm-specific K^+^ channel Slo3 (Stival et al., 2015). This possibility remains to be studied in human sperm where the PKA-dependent activation of CFTR also contributes to the regulation of Em (Puga Molina et al., 2017). In human sperm, then, it is postulated that hyperpolarization may occur as a result of either the increase of K^+^ permeability and/or the reduction of Na^+^ permeability (Santi et al., 2010).

#### K^+^ channels SLO1 and SLO3

In mammalian sperm, it has been shown that hyperpolarization associated with capacitation is inhibited using blockers such as Ba^2+^, which inhibits the inward rectifying K^+^ currents, and the sulfonylureas (tolbutamide and glibenclamide) that inhibit K^+^ channels regulated by ATP (Muñoz-Garay et al., 2001; Acevedo et al., 2006). In addition, it has been reported that the physiological hyperpolarization induced during capacitation in mouse sperm does not depend on the reduction of Na^+^ permeability, but on the increase in K^+^ permeability (Chávez et al., 2013). Two members of the Slo family of K^+^ channels were proposed to have a role in this phenomenon: Slo1, which is highly conserved and ubiquitously expressed, and sperm-specific Slo3, which is present only in mammals and has low sequence conservation (Santi et al., 2010; Miller et al., 2015). As K^+^ currents in mouse sperm depends on the increase in pHi and Slo3 is activated by alkalinization of the cytoplasm, this channel was proposed to be a key player of Em changes in these species (Schreiber et al., 1998; Santi et al., 2010; Zeng et al., 2011). Taking into account that male KO mice for SLO3 are infertile (Santi et al., 2010; Zeng et al., 2011), it is suggested that SLO3 would be the main channel that mediates hyperpolarization in this species.

In human sperm, the participation of K^+^ channels is not as well established as it is in mouse sperm (Kaupp and Strünker, 2016). Human sperm K^+^ current (KSper) is less sensitive to pH and more sensitive to [Ca^2+^]_i_ (Mannowetz et al., 2013), and is inhibited by progesterone (Mannowetz et al., 2013; Brenker et al., 2014). In human sperm, SLO1 was detected by Western blotting (Mannowetz et al., 2013), whereas SLO3 was detected by Western blotting and mass spectrometry (Brenker et al., 2014; López-González et al., 2014). From the biophysical and pharmacological properties that were described with respect to KSper, the currents seem to resemble SLO1 as it is a K^+^ channel activated by Ca^2+^ (Mannowetz et al., 2013). However, recently, it was suggested that capacitated human sperm possess a different type of SLO channel (Mansell et al., 2014) or even a version of SLO3 that is sensitive to Ca^2+^ and weakly dependent on pH (Brenker et al., 2014). It was also proposed that *SLO3* is rapidly evolving in humans, and the variant allele C382R, which is present at a high frequency in the human population, has enhanced apparent Ca^2+^ and pH sensitivities (Geng et al., 2017).

Regarding the participation of SLO3 and SLO1 in male fertility, Brown and coworkers found in a recent study, where 81 subfertile patients undergoing IVF were investigated, that outward K^+^ conductance from these patients was not significantly different from donor sperm. In approximately 10% of the patients, either a negligible outward conductance or an enhanced inward current causing depolarization of Em was observed. Interestingly, in this study, sperm from one patient with low fertilization rate at IVF had very low outward K^+^ conductance and presented depolarized Em. However, no genetic abnormalities in *SLO1, SLO3*, or *LRCC52* genes were found in this patient (Brown et al., 2016).

Regarding the role of SLO1 and SLO3 during capacitation, López-González and coworkers have shown that human sperm capacitated in the presence of inhibitors of both SLO1 and SLO3 have a similar Em to that of sperm incubated in a noncapacitating medium (López-González et al., 2014). Therefore, further investigation is needed to establish the participation of SLO1 and SLO3 in the regulation of Em in human sperm.

#### Na^+^ transport

Previous evidence indicates that Na^+^ participates in establishing the resting Em in mouse sperm (Demarco et al., 2003; Hernández-González et al., 2006). It was also observed in mouse sperm that in an Na^+^-free medium, the addition of this cation induces a rapid depolarization of the Em, which is blocked by EIPA, an analog of amiloride (Escoffier et al., 2012). Both amiloride and EIPA are pharmacological inhibitors of the Na^+^ epithelial channels (ENaC).

ENaC is an heteromultimeric channel composed of the combination of α, β, γ, or δ subunits (de la Rosa et al., 2000; Kellenberger and Schild, 2002). The activity of ENaC channels is closely associated with CFTR, as this channel negatively regulates ENaC (Kunzelmann, 2003; Guggino and Stanton, 2006; Berdiev et al., 2009). In humans, ENaC dysfunction can cause cystic fibrosis among other diseases (Fambrough and Benos, 1999; Snyder, 2002).

In mouse sperm, ENaC-α and ENaC-δ subunits were detected by Western blotting (Hernández-González et al., 2006). In addition, patch-clamp records in testicular sperm detected an amiloride-sensitive component that is in agreement with the presence of ENaC (Martínez-López et al., 2009). In humans, it was demonstrated the presence of the ENaC-δ subunit in the testis (Waldmann et al., 1995) of ENaC-α in the mid-piece of the sperm flagellum by immunocytochemistry and Western blotting (Kong et al., 2009) and the expression of ENaC-β by Western blotting in human sperm (Puga Molina et al., 2018). Interestingly, Kong and coworkers showed that the treatment of human sperm with EIPA improves sperm motility in both healthy donors and asthenozoospermic patients. Puga Molina and coworkers also showed that ${\text{HCO}}_{3}^{-}$ produced a rapid membrane hyperpolarization mediated by CFTR-dependent closure of ENaC channels, which contribute to the regulation of Em during capacitation. In addition, the same authors showed that 1 μM amiloride produces hyperpolarization of the human sperm plasma membrane and decreases [Na^+^]i (Puga Molina et al., 2018).

As mentioned above, previous evidence indicates that mouse and human sperm display a ${\text{HCO}}_{3}^{-}$ uptake through electrogenic Na^+^/${\text{HCO}}_{3}^{-}$ cotransporters (NBC), resulting in a rapid hyperpolarization (Demarco et al., 2003; Puga Molina et al., 2018).

#### Na^+^/K^+^ ATPase

The Na^+^/K^+^ pump is an electrogenic transmembrane ATPase that catalyzes Na^+^ and K^+^ transport by using the energy derived from ATP hydrolysis (Skou, 1957). The proper function of Na^+^/K^+^ ATPase is of vital importance in every cell because it generates the electrochemical gradient for Na^+^ and K^+^ across the plasma membrane (Morth et al., 2007). Na^+^/K^+^ ATPase is an oligomer formed by two subunits: a catalytic α-subunit that contains the sites for binding of Na^+^, K^+^, ATP, and ouabain (an inhibitor of the pump) (Jorgensen et al., 2003), and a β-subunit that is required for guiding the α-subunit to the membrane and for occlusion of the K^+^ ions (Lutsenko and Kaplan, 1993; Geering, 2001). There are several Na^+^/K^+^ ATPase isoenzymes due to the fact that there are 4 different α-subunits (α1, α2, α3, and α4) and 3 different β-subunits (β1, β2, and β3) (Blanco and Mercer, 1998). Each combination is cell- and tissue-specific and displays a particular pattern of expression (Jewell et al., 1992).

The α4-subunit is the most divergent (Woo et al., 2000; Clausen et al., 2016) and is specifically expressed in germ cells of rat, mouse, and human mature sperm (Woo et al., 2000; Sanchez et al., 2006; McDermott et al., 2012; Mcdermott et al., 2015). This isoform is more sensitive to ouabain (Blanco and Mercer, 1998; Sanchez et al., 2006) and is twofold more active than the Na^+^/K^+^ ATPase α1-subunit; which is also expressed in mature rat and human sperm (Shamraj and Lingrel, 1994; Wagoner et al., 2005; Sanchez et al., 2006). Jimenez and coworkers also reported that KO mice that lack α4 are completely sterile. This deletion hindered sperm motility and hyperactivation (Jimenez et al., 2012). Sperm from α4 null-mice showed a depolarized Em due to high [Na^+^]_i_. The fact that the α1 was unable to compensate the absence of α4 demonstrates the absolute requirement of the α4 Na^+^/K^+^ ATPase subunit in mouse sperm fertility (Jimenez et al., 2012). Jimenez and coworkers suggested that the ion gradients maintained by α4 are important for controlling sperm cytoplasmic ion homeostasis because depolarization of the sperm plasma membrane and [Na^+^]i levels are required for sperm motility during sperm capacitation (Jimenez et al., 2012).

It has been reported in rats that during capacitation, α4 Na^+^/K^+^ ATPase increases its activity, resulting in a rise in [K^+^]_i_, a decrease in [Na^+^]_i_, and consumption of ATP (Jimenez et al., 2012). In the same study, the authors showed a higher abundance of α4 in the plasma membrane after the occurrence of capacitation.

Human sperm treated with ouabain showed an [Na^+^]i increase at concentrations that inhibit Na^+^/K^+^ ATPase α1 and α4 (Puga Molina et al., 2018) and a decrease in sperm motility at concentrations that selectively inhibited Na^+^/K^+^ ATPase α4 (Sanchez et al., 2006). McDermott and coworkers studied the function of human Na^+^/K^+^ ATPase α4 in transgenic mice and found higher levels of hyperactive motility compared to wild-type mice, without any alteration in Em or AE (Mcdermott et al., 2015). Therefore, α4 Na^+^/K^+^ ATPase is a very interesting target for male contraception due to its specific localization in sperm and its effects on motility, and its ability to regulate intracellular Na^+^ and K^+^.

## Calcium requirements during capacitation

Sperm functional changes that take place during capacitation depend on a combination of sequential and concomitant signaling processes (Visconti et al., 2011), which includes complex signaling cascades where intracellular Ca^2+^ plays a central role. There are some reports where Ca^2+^ levels were measured and showed an increase in intracellular Ca^2+^ concentration ([Ca^2+^]_i_) during mammalian sperm capacitation (Jai et al., 1978; Coronel and Lardy, 1987; White and Aitken, 1989; Ruknudin and Silver, 1990; Zhou et al., 1990; Baldi et al., 1991; Cohen et al., 2014; Luque et al., 2018). Moreover, the importance of this ion in the regulation of sperm motility, hyperactivation, and AE has been demonstrated through several pharmacological and genetic loss-of-function approaches (Suarez and Dai, 1995; Ho and Suarez, 2001; Darszon et al., 2011).

It has been described that Ca^2+^ can directly bind to membrane phospholipids and to numerous enzymes, modifying the membrane properties and enzymatic activity. This ion may also bind to calmodulin (CaM), and CaM antagonists have been shown to inhibit certain aspects of sperm function, as hyperactivated motility (Si and Olds-Clarke, 2000). Ca^2+^ binding to CaM causes conformational changes, and this complex modulates the activity of adenylyl cyclases (Gross et al., 1987), phosphatases (Tash et al., 1988; Rusnak and Mertz, 2000), phosphodiesterases (Wasco and Orr, 1984), and protein kinases (Hook and Means, 2001; Marín-Briggiler, 2005). Interestingly, testis specific ADCY10 is Ca^2+^-dependent but CaM-independent (Jaiswal and Conti, 2003; Litvin et al., 2003), suggesting that Ca^2+^ regulates capacitation through multiple pathways. In particular, it has been shown in sperm of marine invertebrates that rises in [Ca^2+^]_i_ modulate the sperm swimming behavior by changing the flagellar beat pattern through Ca^2+^-sensing proteins, calaxins (Mizuno et al., 2009, 2012). Dynein activity is inhibited within the axoneme by Ca^2+^-bound calaxins, resulting in the high-amplitude asymmetric flagellar bending—typical of hyperactivated motility (Shiba et al., 2008).

As detailed above, one of the first events that triggers sperm capacitation is the activation of a cAMP pathway (Buffone et al., 2014b). At ejaculation, human sperm interact with higher ${\text{HCO}}_{3}^{-}$ and Ca^2+^ concentrations present in the seminal fluid (Homonnai et al., 1978; Okamura et al., 1985). This causes an increase in cAMP levels by the opposing activities of the ADCY10 and PDE that stimulates PKA-dependent phosphorylation of proteins in Ser/Thr residues (Osheroff et al., 1999; Visconti et al., 2011; Battistone et al., 2013). Evidence in mouse and human sperm has shown that PKA-dependent phosphorylation is also regulated by the Src family kinase (SFK) inactivation of Ser/Thr phosphatases (Krapf et al., 2010; Battistone et al., 2013). Phosphorylation of PKA substrates leads to pY in sperm of all mammalian species studied (Visconti et al., 1995b; Leclerc et al., 1996; Galantino-Homer et al., 1997; Osheroff et al., 1999). Genetic loss-of-function experiments in mice demonstrated the essential role of proteins involved in the cAMP pathway in sperm capacitation and fertilization (Hess et al., 2005). On the other hand, mouse sperm exposed to the Ca^2+^ ionophore A23187 are able to develop hyperactivation, undergo AE, and acquire fertilizing ability even when the cAMP pathway is completely abolished (Tateno et al., 2013).

Ca^2+^ requirements during mammalian sperm capacitation have been widely studied in the murine and human models. Incubation of mouse sperm in the absence of added extracellular Ca^2+^ prevented the capacitation-associated increase in pY (Visconti et al., 1995a). However, the addition of EGTA to further lower the extracellular Ca^2+^ (medium without added Ca^2+^ still contains micromolar concentrations of this cation) promotes a strong increase in pY. A similar effect was also observed when adding CaM antagonists or calcineurin inhibitors (Navarrete et al., 2015). These results led the authors to propose that Ca^2+^ modulates mouse sperm cAMP and pY pathways in a biphasic manner, having both positive and negative roles, and that some of its effects are mediated by CaM (Navarrete et al., 2015). Recent studies have shown that in mouse sperm, the tyrosine kinase FER is involved in the capacitation-associated increase in pY (Alvau et al., 2016). Interestingly, human sperm display a different type of Ca^2+^ regulation during capacitation. Several reports have shown that extracellular Ca^2+^ negatively modulates phosphorylation on tyrosine residues, as human sperm incubated in a medium without added Ca^2+^ displayed increased pY compared to those incubated in complete medium (Carrera et al., 1996; Leclerc and Goupil, 2002; Marín-Briggiler et al., 2003; Baker et al., 2004; Battistone et al., 2014). The lack of added Ca^2+^ in the medium would lead to an increased tyrosine kinase activity through higher levels of ATP (Baker et al., 2004). In human sperm, lowering extracellular [Ca^2+^] was accompanied by a decrease in both ADCY10 activity and cAMP levels (Jaiswal and Conti, 2003; Torres-Flores et al., 2011), without affecting PDE1 activity (Lefièvre et al., 2002) and PKA-mediated phosphorylation (Battistone et al., 2014). Inhibition of CaM also increased pY with no changes in PKA-mediated phosphorylation, supporting the role of CaM in the increase in pY observed without adding Ca^2+^ to the medium (Battistone et al., 2014). As previously mentioned, PYK2 has been identified as the Ca^2+^-dependent kinase involved in human sperm pY downstream PKA activation (Battistone et al., 2014).

Regarding Ca^2+^ requirements to maintain human sperm function *in vitro*, it has been reported that 0.22 mM Ca^2+^ is sufficient for the development of pY and hyperactivated motility, whereas more than 0.58 mM of this cation is necessary to maintain follicular fluid-induced AE and sperm–ZP interaction (Marín-Briggiler et al., 2003). Therefore, transit through the female tract affords sperm to be modified by changes in the ionic environment that are not available in *in vitro* models. Moreover, there is evidence indicating that Ca^2+^ ions can be replaced by Sr^2+^ in maintaining human sperm capacitation-related events (Marín-Briggiler et al., 1999). These results would indicate that at least *in vitro*, different sperm events have specific Ca^2+^ requirements. Such information can be used for the development of culture conditions that would allow the dissociation of these events of the fertilization process.

### Calcium transport systems in sperm

Some aspects of sperm physiology depend on the maintenance and regulation of [Ca^2+^]_i_, which involves a range of pumps and channels at the plasma membrane or intracellular stores that import, export, and/or sequester Ca^2+^ ions [reviewed by (Jimenez-Gonzalez et al., 2006; Clapham, 2007; Darszon et al., 2007, 2011; Correia et al., 2015)].

Two Ca^2+^ transport systems have been identified in mammalian sperm. The first one involves Ca^2+^ efflux through the plasma membrane Ca^2+^ ATPase (PMCA) and the Na^+^/Ca^2+^-exchanger (NCX), which pump Ca^2+^ out of the cell or into intracellular Ca^2+^ stores (Michelangeli et al., 2005). PMCA, localized in the principal piece of the flagellum of mouse sperm, is relevant for sperm function as its ablation alters sperm motility. Sperm from *PMCA4b* KO mice failed to develop hyperactivated motility and therefore are sterile (Okunade et al., 2004; Schuh et al., 2004). Mitochondrial abnormalities found in PMCA4-deficient sperm (Okunade et al., 2004) suggest Ca^2+^ overload due to defective Ca^2+^ extrusion. NCX is present in the plasma membrane of mammalian sperm (Babcock and Pfeiffer, 1987) and is thought to be of great importance for the regulation of Ca^2+^ homeostasis (Reddy et al., 2001; Su and Vacquier, 2002). Pharmacological inhibition of NCX provokes an increase in [Ca^2+^]_i_ and a significant reduction of human sperm motility (Krasznai et al., 2006).

The second Ca^2+^ transport system is related to Ca^2+^ influx and involves mainly the sperm-specific Ca^2+^ channel CatSper (see below). Other Ca^2+^ plasma membrane channels have also been identified in spermatogenic and sperm cells. Several voltage-gated Ca^2+^ (Ca_v_) channel subunits have been detected in the head and flagellum of mammalian sperm, and their activity has been assessed in both germ cells and sperm (Arnoult et al., 1996, 1999; Serrano et al., 1999; Westenbroek and Babcock, 1999; Wennemuth et al., 2000; Sakata et al., 2002; Cohen et al., 2014). In particular, animals devoid of the α_1E_ subunit of the Ca_v_2.3 channel show aberrant sperm motility (Sakata et al., 2002) and reduced litter sizes and IVF success, mainly due to impaired ability to undergo AE (Cohen et al., 2014). It has been suggested that the interaction of sperm Ca_v_2.3 channel subunits with membrane G_M1_ regulates Ca^2+^ currents and the occurrence of AE (Cohen et al., 2014). In addition, T-type Ca_v_3 channel subunits have been found in the head and flagellum of mouse and human sperm; however, drugs that inhibit these channels do not affect human sperm motility (Treviño et al., 2004). Cyclic nucleotide-gated channels (CNG), permeable to Ca^2+^, have also been described in bovine testis and sperm and suggested to be involved in sperm motility (Wiesner et al., 1998). The A subunit was observed along the flagellum, whereas the short B subunit is restricted to the principal piece. Furthermore, there is evidence showing that CNG channels act as a Ca^2+^ entry pathway being more responsive to cGMP rather than to cAMP (Wiesner et al., 1998). The A3 and B1 subunits are present in the flagellum of mouse sperm, but the A3 null mice are fertile (Kaupp and Seifert, 2002), questioning the relevance of these channels in sperm physiology. Moreover, some members of the transient receptor potential channel (TRPC) family have been found in the flagella of mouse (transient receptor potential canonical; TRPC1 and C3) (Treviño et al., 2001) and human sperm (TRPC1, C3, C4, and C6), and their inhibition abolished human sperm motility (Castellano et al., 2003). More recently, store-operated channel proteins (ORAI) and their activators, i.e., STIM, have been shown to interact with TRPC and regulate sperm function (Darszon et al., 2012).

Sperm intracellular Ca^2+^ can be exchanged to or from internal stores localized in the acrosome, as well as in the neck (redundant nuclear envelope, RNE) by inositol triphosphate and ryanodine receptors (IP_3_R and RyR, respectively) (Darszon et al., 2011; Visconti et al., 2011). In human sperm, the presence of the RyR was determined by several techniques and it was located mainly in the neck region and very rarely in the acrosome (Harper et al., 2004; Lefièvre et al., 2007), whereas the IP3 receptors were found in the neck region and in the acrosome (Dragileva et al., 1999; Kuroda et al., 1999; Rossato et al., 2001). In the acrosome, it was demonstrated that Ca^2+^ is mobilized through the IP-3-sensitive channel (De Blas et al., 2002; Branham et al., 2009; Lopez et al., 2012), and it is proposed that the Ca^2+^ influx by these channels is dependent on ${\text{HCO}}_{3}^{-}$ and involves EPAC activity. It has been shown that Ca^2+^ release from the reservoirs is a necessary event for the AE (De Blas et al., 2005), and that hyperactivated motility depends on the mobilization of intracellular Ca^2+^ by IP_3_R activation (Alasmari et al., 2013), and chemotaxis in response to progesterone needs Ca^2+^ mobilization from intracellular stores followed by the activation of TRPC (Teves et al., 2009). In addition, two Ca^2+^ pumps were identified and located in human sperm: sarcoplasmic-endoplasmic reticulum Ca^2+^ ATPase (SERCA) (Lawson et al., 2007) and secretory pathway Ca^2+^ ATPases (SPCA) (Harper et al., 2005). Both are sensitive to thapsigargin at different concentrations (Thastrup et al., 1990; Rossato et al., 2001; Harper et al., 2005). SERCA 2 has been localized in the acrosome and mid-piece regions of mammalian species, including human, and it has been suggested to participate in Ca^2+^ sequestration in internal stores during sperm capacitation (Lawson et al., 2007).

### CatSper channel: structure and regulation

Despite a large body of evidence indicating the presence of multiple Ca^2+^ channels in human sperm, their activity has not been totally elucidated. The advent of sperm electrophysiology (Kirichok et al., 2006; Lishko et al., 2010) allowed the characterization of Ca^2+^ currents through CatSper channels. This channel complex is localized in the sperm flagellum and comprises four homologous α subunits (CatSper 1–4) (Navarro et al., 2008; Kirichok et al., 2011) and auxiliary subunits: CatSper β, CatSper ɤ, and CatSper δ (Liu et al., 2007; Wang et al., 2009; Chung et al., 2011). Deficiency of any subunit affects the expression of all the other subunits and is detrimental to male fertility (Qi et al., 2007). Recently two new auxiliary subunits have been described: CatSper ξ and CatSper ζ (Chung et al., 2017). Evidence from KO mice has shown that CatSper is essential for hyperactivation and fertilization (Ren et al., 2001; Quill et al., 2003). CatSper-KO sperm are unable to migrate efficiently *in vivo* (Ho et al., 2009; Chung et al., 2014) and penetrate the egg cumulus (Chung et al., 2017) and the ZP (Ren et al., 2001). However, a transient exposure to Ca^2+^ ionophore A23187 enables *in vitro* fertilization of these as well as other KO sterile mice models (Navarrete et al., 2016). In contrast to what occurs in wild-type sperm, CatSper1 KO undergoes PKA activation and a remarkable increase in pY even in nominal zero Ca^2+^ media, suggesting that CatSper transports the Ca^2+^ involved in the regulation of the cAMP-PKA-dependent pathway required for sperm capacitation (Navarrete et al., 2015). Moreover, evidence in the human has shown that point mutations within the CatSper1 gene as well as deletion of the CatSper2 gene are related to male infertility (Avidan et al., 2003; Zhang et al., 2007; Hildebrand et al., 2010). In a CatSper2-deficient infertile patient, no appreciable CatSper current was observed, which is caused by the complete lack of other CatSper complex members (Smith et al., 2013).

Recent groundbreaking work from Chung and coworkers using super-resolution microscopy (STORM) in the mouse model showed that CatSper distributes longitudinally following four backbone lines, which are localized in the plasma membrane of the principal piece, close to the fibrous sheath (Chung et al., 2014, 2017). Similarly, it has been reported that human CatSper ξ is arranged in four domains along the flagellum (Chung et al., 2017). Together with CatSper, other signaling molecules display a similar spatial distribution along the principal piece, which reveals a complex organization of signaling pathways in the sperm flagellum that focuses pY in time and space (Chung et al., 2014). In addition, a variation in subflagellar localization of CatSper domains in capacitated sperm has been described by 3D STORM (Chung et al., 2014). It has been reported that approximately 30% of sperm presented a quadrilateral CatSper1 domain organization and they were able to display hyperactivated motility and pY (Chung et al., 2014). This is consistent with the observations made by different groups that only a subpopulation of sperm achieved hyperactivation upon capacitation (Kulanand and Shivaji, 2001; Buffone et al., 2009a; Goodson et al., 2011). However, evidence in human sperm suggests that the development of hyperactivation does not directly depend on CatSper activation, but on the release of stored Ca^2+^ at the sperm neck (Alasmari et al., 2013). It has been proposed that CatSper channels would rather be involved in intracellular Ca^2+^ stores mobilization during sperm capacitation, affecting hyperactivated motility indirectly (Alasmari et al., 2013).

CatSper activity was directly recorded in 2006 using the patch-clamp method (Kirichok et al., 2006). Murine CatSper current (*I*_CatSper_) is weakly voltage dependent (Kirichok et al., 2006), which can be attributed to heterogeneity in the arginine and lysine compositions of the putative voltage sensor domains from each CatSper α subunits. Mice devoid of any CatSper α subunits or the CatSper δ do not display the *I*_CatSper_ and, as previously mentioned, are all infertile (Carlson et al., 2003, 2005; Kirichok et al., 2006; Liu et al., 2007; Qi et al., 2007; Wang et al., 2009; Chung et al., 2011). Human *I*_CatSper_ was recorded in 2010 (Lishko and Kirichok, 2010; Lishko et al., 2010), and the comparison with mouse CatSper currents revealed important differences in this channel regulation and function. The human CatSper channel is slightly more voltage dependent in comparison to the mice one. Although intracellular alkalinization allows the opening of mice CatSper channels, this is not sufficient for human sperm (Kirichok et al., 2006; Lishko et al., 2010, 2011). The highly enriched histidine composition of the N-termini of both CatSper1 proteins is thought to be involved in the pH sensitivity of the channel, which made this difference totally unexpected.

In addition to voltage and pHi, mammalian CatSper is also controlled by numerous ligands present in the oviductal fluid as well as different synthetic chemicals (Kirichok et al., 2006; Lishko et al., 2010, 2011; Strünker et al., 2011; Brenker et al., 2012; Tavares et al., 2013). In humans but not mice, progesterone (Lishko et al., 2011; Strünker et al., 2011) activates CatSper via binding to the serine hydrolase ABHD2 (α/β hydrolase domain–containing protein 2) (Miller et al., 2016; Mannowetz et al., 2017). It has been shown that at rest the human CatSper channel is inhibited by the endocannabinoid 2-arachidonoylglycerol (2-AG); after progesterone binding, ABHD2 degrades 2-AG, relieving CatSper inhibition (Miller et al., 2016). The Ca^2+^ influx mediated by progesterone has been involved in sperm capacitation, chemotaxis, hyperactivation, and AE (Harper et al., 2003; Oren-Benaroya et al., 2008; Publicover et al., 2008), but the participation of CatSper has been unequivocally demonstrated in hyperactivation. Prostaglandins also activate the human CatSper channel, but independent of the ABHD2 mechanism (Miller et al., 2016). The prostaglandins-induced Ca^2+^ influx evokes AE and increases motility (Aitken and Kelly, 1985; Schaefer et al., 1998). Both progesterone and prostaglandin modulation is suggested to be restricted to human and primate sperm (Lishko et al., 2011) and do not involve classical nuclear receptors or G protein-coupled receptors (GPCRs) (Lishko et al., 2011; Strünker et al., 2011).

More recently, patch-clamp recordings from human sperm revealed that the neurosteroid pregnenolone sulfate exerted similar effects as progesterone on CatSper currents (Mannowetz et al., 2017; Brenker et al., 2018). CatSper-deficient patients were described as infertile (Avidan et al., 2003; Zhang et al., 2007). Further studies showed that these sperm did not produce any progesterone (Smith et al., 2013) nor pregnenolone sulfate-activated currents (Brenker et al., 2018).

Nowadays, there are some controversies about the effects of testosterone, estrogen, and hydrocortisone on CatSper currents. Results from Mannowetz and coworkers revealed that they abolish CatSper activation by progesterone but these steroids do not activate CatSper themselves (Mannowetz et al., 2017). On the other hand, more recent evidence from Brenker et al. determined that testosterone, hydrocortisone, and estradiol are agonists that activate CatSper (Brenker et al., 2018). These differences might be due to different conditions and experimental approaches. Further studies are needed to understand this complex regulation.

The mechanism underlying the activation of the CatSper channel by various ligands remains largely unknown. There are reports that suggest the involvement of human ß-defensin 1, a small secretory peptide with antimicrobial activities, which interacts with the sperm chemokine receptor type 6 (CCR6), triggering Ca^2+^ mobilization (Com et al., 2003; Caballero-Campo et al., 2014; Diao et al., 2014). CCR6 colocalizes and interacts with CatSper in human sperm, and both CCR6 and CatSper are required for the Ca^2+^ entry/current induced by physiological ligands DEFB1, chemokine (C-C motif) ligand 20 (CCL20), and progesterone in human sperm (Diao et al., 2017). Environmental toxins, including some endocrine disruptors, have also been shown to induce the [Ca^2+^]_i_ increase through CatSper activation (Tavares et al., 2013; Schiffer et al., 2014). In addition, previous reports have shown that bovine serum albumin (BSA) induces [Ca^2+^]_i_ influx through CatSper channel activation (Xia and Ren, 2009), because this response is absent in CatSper1 KO sperm. Lishko and coworkers suggested that the modification in the lipid content of the sperm plasma membrane may induce CatSper gating (Lishko et al., 2012), as albumin was reported to induce a Ca^2+^ influx through CatSper in mouse sperm (Xia and Ren, 2009). This possibility remains to be elucidated.

The characterization of CatSper function and regulation encounters several difficulties due to: (i) the promiscuous nature of CatSper activation, (ii) the lack of specific antagonists, and (iii) so far, CatSper expression in heterologous systems has not been possible. Regarding the regulation of CatSper during capacitation, evidence in the mouse sperm suggests that SLO3 K^+^ channels control Ca^2+^ entry through CatSper (Chávez et al., 2014). High concentrations of ${\text{HCO}}_{3}^{-}$ trigger an initial change in the pHi, which activates SLO3 channels (Santi et al., 2010); the resulting membrane hyperpolarization raises pHi even more, probably through an NHE mechanism (Chávez et al., 2014). This intracellular alkalization activates the CatSper channel, which results in a very rapid [Ca^2+^]_i_ increase. On the other hand, Ca^2+^ rather than pHi controls KSper in human sperm (Mannowetz et al., 2013). Therefore, it was suggested that CatSper might be placed upstream SLO1/3 (Kaupp and Strünker, 2016). In this regard, a recent report indicated that certain patients show impaired K^+^ conductance and abnormal resting Em, but normal resting [Ca^2+^]_i_ and progesterone-induced [Ca^2+^]_i_ responses similar to those of donor sperm, which suggest unaltered CatSper function (Brown et al., 2016). However, further studies are needed to determine the relationship among sperm ion channels and to establish the similarities and differences between mouse and human sperm.

## Final remarks

This review is focused on the principal molecular mechanisms that govern human sperm capacitation with particular emphasis in comparing all the reported evidence with the mouse model.

**Figure 1:**
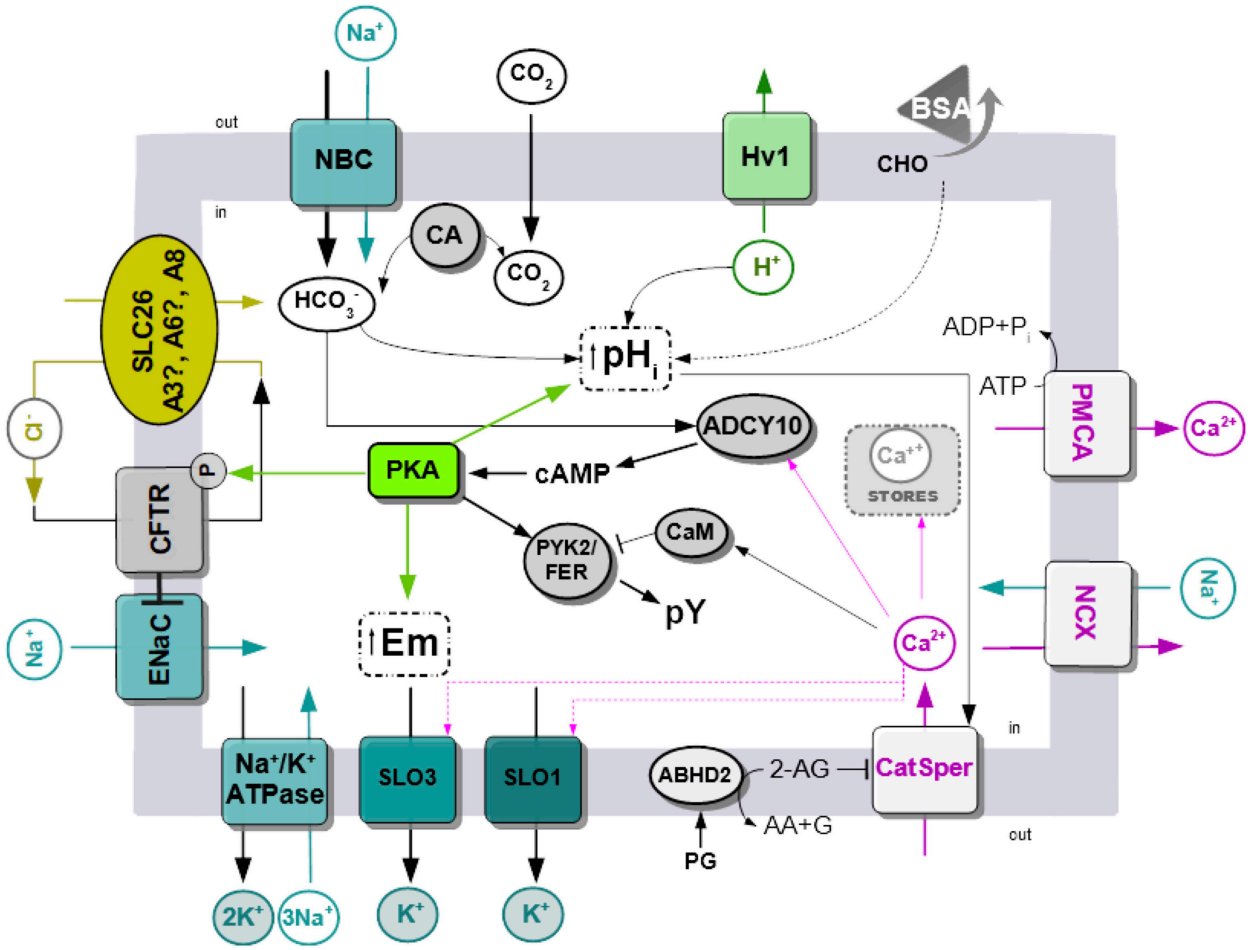
Simplified model of signaling pathways an ion fluxes involved in human sperm capacitation. Na^+^/K^+^ ATPase, Na^+^/K^+^ pump ATPase; SLO1 and 3, sperm-specific K^+^ channel 1 and 3; ENaC, epithelial Na^+^ channels; CFTR, cystic fibrosis transmembrane conductance channel; SLC26, solute carrier 26, there is still no evidence of A3 and A6 is present in mature human sperm; Hv1, voltage-gated H^+^ channels; BSA, bovine serum albumin; CHO, cholesterol; CA, carbonic anhydrase; PYK2/FER, proline-rich tyrosine kinase 2; ADCY10, atypical soluble adenylyl cyclase 10; EPAC, exchange protein activated by cAMP; CaM, calmodulin; CatSper, sperm-specific Ca^2+^ channel; NCX, Na^+^/Ca^2+^-exchanger; PMCA Plasma Membrane Ca^2+^ ATPase; PG, progesterone; ABDH2, α/β hydrolase domain–containing protein 2; 2-AG, 2-Arachidonoylglycerol; AA, arachidonic acid; G, glycerol.

The data presented in this review are summarized in Figure 1. Sperm are exposed to higher ${\text{HCO}}_{3}^{-}$ concentration at the time of ejaculation and during their transit through the female reproductive tract. In addition, removal of sperm cholesterol from the plasma membrane to acceptors present in the uterus and fallopian tubes, such as albumin, results in biophysical modification of the plasma membrane. The best characterized change is the increase in membrane fluidity. The initial ${\text{HCO}}_{3}^{-}$ transport through NBC cotransporters activates ADCY10 and that in turn produce an increase in cAMP concentration, leading to the activation of PKA. Phosphorylation by PKA is essential for CFTR activity, and together with other Cl^−^/${\text{HCO}}_{3}^{-}$ cotransporters (SLC A3/6/8), it produces a sustained increase in ${\text{HCO}}_{3}^{-}$. Other possible sources of ${\text{HCO}}_{3}^{-}$ may be related to the action of carbonic anhydrases. Activation of PKA led to protein tyrosine phosphorylation by mechanisms that are not completely elucidated, which involved the kinases PYK2/FERT. At the same time, upon contact with ${\text{HCO}}_{3}^{-}$, there is an increase in sperm intracellular pH. Human sperm alkalinization is also favored by the efflux of proton through Hv1 channels. Alkalinization and certain steroids present in the female reproductive tract such as progesterone activate CatSper channels and produce a sustained increase in [Ca^2+^]i. The levels of [Ca^2+^]i are also regulated by the action of exchangers and pumps such as NCX and PMCA. Activation of cAMP/PKA pathways also leads to hyperpolarization of the plasma membrane. The contribution of both the opening of K^+^ channels (SLO1 and/or SLO3) and the closure of Na^+^ channels, such as ENaC, was reported. In the last case, ENaC is inhibited by CFTR.

These complex molecular mechanisms built over the time using results from different groups do not take into account the following two important considerations:

- In the mouse, the estrous (receptive period) lasts less than a day, and mating is timed to favor the encounter between sperm and eggs. However, in humans, the timing of when sperm encounter the egg is spread over a long period of time (2–3 days), and concomitantly, human sperm require long incubation time (more than 3 h) to undergo capacitation-related events. In this regard, it was recently demonstrated that the timing of human sperm capacitation (as evaluated with the novel Cap-score™) is reproducible within each individual but varies among men (Ostermeier et al., 2018). The Cap-Score™ was defined as the percentage of sperm having G_M1_ localization patterns consistent with capacitation and was shown to be a good indicator of male fertility (Cardona et al., 2017). The reasons for such variability are unknown but they may be related with the fact that human semen samples are not homogeneous. They are composed of different subpopulations of sperm with different functional features (Buffone et al., 2004).
- All the *in vitro* experiments conducted so far to study capacitation were performed in the absence of the periovulatory female reproductive tract. For example, the occurrence of AE was long enough to occur upon sperm binding to the ZP. However, recent evidence from different laboratories demonstrated *in vivo* that mouse sperm undergo AE prior to encountering the cumulus-oocyte complexes in the upper segments of the oviduct (Hino et al., 2016; La Spina et al., 2016; Muro et al., 2016). However, this is not the case for human sperm because *in vivo* experimentation using human sperm is virtually impossible nowadays for ethical consideration. Therefore, immediate translation of previous capacitation investigations from mouse to human is questionable and must be analyzed with caution. In addition, the high success rates of ICSI also had a negative impact on basic reproductive studies in both humans and mice. However, this is not the case for artificial insemination where pregnancy rates are really low. The understanding of molecular mechanisms underlying human sperm capacitation would help in the treatment of patients subjected to low-complexity assisted fertilization procedures, but also, it is essential to the development of alternative contraceptive strategies.

## Author contributions

MB defined the topics. LP, GL, PB, CM-B, and MB wrote the paper. All authors discussed the results and implications and commented on the manuscript at all stages.

### Conflict of interest statement

The authors declare that the research was conducted in the absence of any commercial or financial relationships that could be construed as a potential conflict of interest.
